# Alterations of the Erythrocyte Membrane during Sepsis

**DOI:** 10.1155/2012/702956

**Published:** 2012-05-21

**Authors:** Yasmina Serroukh, Sarah Djebara, Christophe Lelubre, Karim Zouaoui Boudjeltia, Patrick Biston, Michael Piagnerelli

**Affiliations:** ^1^Department of Intensive Care, CHU-Charleroi, Université Libre de Bruxelles, 92, Boulevard Janson, 6000 Charleroi, Belgium; ^2^Experimental Medicine Laboratory, CHU-Charleroi, ULB 222 Unit, 6110 Montigny-le-Tilleul, Belgium

## Abstract

Erythrocytes have been long considered as “dead” cells with transport of oxygen (O_2_) as their only function. However, the ability of red blood cells (RBCs) to modulate the microcirculation is now recognized as an important additional function. This capacity is regulated by a key element in the rheologic process: the RBC membrane. This membrane is a complex unit with multiple interactions between the extracellular and intracellular compartments: blood stream, endothelium, and other blood cells on the one hand, and the intracytoplasmic compartment with possible rapid adaptation of erythrocyte metabolism on the other. In this paper, we review the alterations in the erythrocyte membrane observed in critically ill patients and the influence of these alterations on the microcirculatory abnormalities observed in such patients. An understanding of the mechanisms of RBC rheologic alterations in sepsis and their effects on blood flow and on oxygen transport may be important to help reduce morbidity and mortality from severe sepsis.

## 1. Introduction

The microcirculation, which includes all vessels with a diameter <100 *μ*m, blood cells (red blood cells-RBCs-, white blood cells-WBCs- and platelets), endothelium, and microparticles, plays a central role in tissue oxygenation because it is across the walls of the microvessels that oxygen (O_2_) diffuses from the blood to the cells within each tissue. Alterations at this circulatory compartment level are frequently observed in critically ill patients, especially in those with sepsis [1–5], and persistence of these alterations is associated with a poor outcome [4, 6]. 

**Figure 1:**
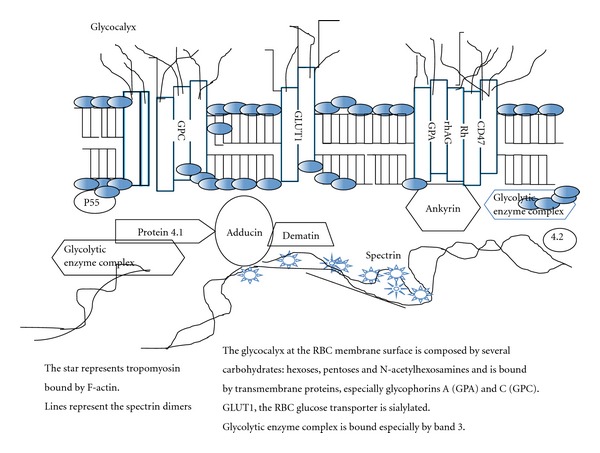
Schematic representation of the red blood cell membrane.

RBCs were formerly considered as a simple container (the membrane) for one important cytoplasmic protein: haemoglobin. The membrane is considered as one of the key determinants of RBC deformability, alongside cell geometry and cytoplasmic viscosity. Understanding the relationship between the different components of the membrane (lipids-50% of molecular weight, proteins- 40%- and carbohydrates- 10%-) (Figure 1) [6–8] helps explain the deformability process. All these components may be altered in sepsis, through a direct effect of bacteria, or via enzymes and/or reactive oxygen species (ROS) produced by WBCs and/or platelets. However, few studies have evaluated membrane alterations during sepsis.

Interestingly, several recent studies have reported an important role of the RBC in modulating the microcirculation. For these reasons, RBCs are no longer considered merely as “dead cells” without a nucleus and only a membrane and haemoglobin to transport O_2_ and CO_2,_ but as living cells capable of modulating the microcirculation in response to various stimuli. 

This review first reports the alterations in RBC membrane components and biochemistry observed during sepsis and, second, the possible contribution of these altered RBCs to the microcirculatory abnormalities observed during sepsis (Table 1 for summary).

## 2. The RBC Membrane

The RBC membrane is considered as a key element in RBC rheology, especially deformability. It is composed of proteins (52% of the molecular weight), lipids (30%), and carbohydrates (8%) with complex interactions among these elements. The membrane and these interactions have been reviewed elsewhere [6–8]. Modifications of RBC membrane components could alter RBC rheology and probably also RBC biochemistry. Here we review the alterations of the RBC membrane that have already been described during sepsis. 

### 2.1. Proteins

Few works have studied modifications of the protein part of the RBC membrane during sepsis. Nieuwland et al. [10] measured, in the sera of patients with meningococcaemia, the concentrations of microparticles derived from leucocytes (granulocytes and monocytes), endothelial cells, and platelets. As controls, these authors measured microparticles containing glycophorin A—a major integral protein of the RBC membrane (Figure 1). These authors observed increased blood concentrations of the different microparticles from WBCs, platelets, and endothelial cells during the first 36 hours of sepsis, but no modifications in glycophorin A. These data suggest that the RBC membrane glycophorin A content remains constant, at least during the early stage of sepsis [10]. 

In a mouse model of septic shock induced by caecal ligation and puncture, Spolarics et al. [11] studied the effects of sepsis on RBC glucose-6-phosphodehydrogenase (G-6-PD) knock-out mice. This enzyme, the first in the pentose phosphate pathway, enables the synthesis of NADPH for glutathione production and antioxidant defenses [8, 18]. These authors observed increased haemolysis in the “knock-out” mice, with significant alterations in RBC deformability. To explain these results, Spolarics et al. [11] hypothesised that the RBC membrane becomes unstable because of an increased band 3/*α*-spectrin ratio, suggesting an alteration of the membrane integral/peripheral protein ratio (Figure 1). Interestingly, this band 3/*α*-spectrin ratio was also increased in the group of septic “sham” mice with decreased deformability but without haemolysis compared to “knock-out” G6PD mice [11]. 

The same authors [12] continued their studies on sepsis and the RBC membrane, especially band 3. In the same animal model, they observed that sepsis induced a significant increased in tyrosine phosphorylation of band 3. This phosphorylation modified the link between band 3 and other proteins of the membrane, but without effects on the anion transport capacity of band 3 [12].

Because of interspecies differences in membrane composition, rheology, and biochemistry, these observations need confirmation in humans. Piagnerelli et al. [13] studied RBC membrane proteins from healthy volunteers and from patients with and without sepsis within 24 hours of ICU admission, and on day 3 for the septic patients. Procedures included screening for alterations in RBC membrane proteins using cryohaemolysis and separation of RBC membrane and skeletal proteins using polyacrylamide gel electrophoresis in the presence of sodium dodecylsulfate. The majority of RBC membrane protein ratios, including band 3/spectrin, were more elevated in critically ill patients (nonseptic and septic) than in volunteers, but RBC membrane skeletal protein content was similar in septic and nonseptic patients [13]. There were no significant differences in cryohaemolysis results among groups. The authors concluded that there were differences in the RBC membrane protein content between critically ill patients within 24 hours of ICU admission and healthy volunteers, but no differences in membrane protein content in septic patients compared to non-septic patients, suggesting that sepsis per se does not alter the RBC membrane protein content.

ROS can also alter the protein part of the membrane and thus the deformability. Uyesaka et al. [19] observed that incubation of RBCs with O_2_^−^ induced a rapid and important degradation of RBC membrane proteins (band 3 and spectrin) with the formation of a new protein band in the membrane. This new organization of the protein part of the membrane may decrease RBC deformability [19].

On the other hand, the RBC membrane participates in ROS synthesis in hypoxic conditions. Kiefmann et al. [20] recently demonstrated in a rat model of isolated and perfused lungs that H_2_O_2_ was produced by RBCs as a result of autooxidation of haemoglobin located on the membrane. RBCs then transported ROS to endothelial cells. In response, endothelial cells increased intracytosol Ca^2+^-inducing P-selectin expression on the plasma membrane favouring leucocyte adhesion in venules and capillaries. All these processes contribute to inflammation [20].

### 2.2. Lipids

The lipid portion of the human RBC membrane may be altered during sepsis. Todd et al. studied, using spectroscopy, the effects of endotoxins on the viscosity of the lipid part of the RBC membrane [21]. They noted an increased viscosity of these lipids without modifications in mean corpuscular volume or mean corpuscular haemoglobin concentration, suggesting no loss of this portion of the RBC membrane [21]. Interestingly, Kempe et al. [14] observed that incubation of RBCs from healthy volunteers with plasma from septic patients induced phosphatidylserine expression on the RBC membrane surface suggested by fixation of annexin V in flow cytometry [14]. These results were identical when they incubated RBCs with the supernatant of bacteria cultures [14]. The physiopathologic mechanism to explain these results, suggested by Kempe et al. [14], is the formation of membrane ceramide, which could induce a significant increase in RBC Ca^2+^ concentrations. This effect in turn stimulates K^+^-Ca^2+^ channels, which, with Cl^−^ channels, drives KCl out of the cells. All of these modifications induce cellular dehydration. Indeed, according to these authors, sepsis induces eryptosis—programmed RBC death—in contrast to apoptosis for nuclear cells [22].

Lipid peroxidation of the RBC membrane also seems to be increased during sepsis. Huet et al. [15] observed this effect by measuring ROS production, using thiobarbituric acid-malondialdehyde solutions, by RBCs from septic patients already on the first day of severe sepsis. In rats with sepsis induced by caecal ligature and perforation, Baskurt et al. also observed significantly increased lipid peroxidation [16], but these results were not confirmed in a study by Bateman et al. [17].

### 2.3. Carbohydrates

Carbohydrates are a minor component of the RBC membrane, representing only 8% of the molecular weight of the human RBC membrane. The RBC glycocalyx (Figure 1) is dominated by the carbohydrate domains of glycolipids and integral glycoproteins. These oligosaccharides contain, in addition to neutral hexoses, pentoses and N-acetylhexosamines, fully ionised sialic acid. Sialic acid (sialon in Greek word: saliva), less commonly called neuraminic acid, is the designation given to a family of over 40 naturally occurring 9-carbon keto sugars acids derived from N-acetylneuraminic acid (Neu5AC) [23]. The most abundant derivative of sialic acid present in humans and in RBCs is N-acetylneuraminic acid [23] (SA). 

Glycophorin A, the most important transmembrane protein, is highly glycosylated, with approximately 60% of its weight attributable to carbohydrates. Most of the carbohydrate is in the form of 15 O-glycosidically linked tetrasaccharides. The two SA residues of each of these many O-glycosidically linked oligosaccharides account for 60% to 90%, depending on the species, of the negative charge of the RBC membrane surface [24] and account for the fact that RBCs normally repel each other and do not aggregate [6, 7].

Piagnerelli et al. [9] observed, already within the first 24 hours of ICU admission, a significant inverse relationship between RBC shape, assessed by a flow cytometry technique, and the RBC membrane SA content in critically ill patients with and without sepsis. They observed a more spherical shape in RBCs from septic compared to non-septic patients and healthy volunteers, associated with a decreased RBC membrane SA content. To exclude the possible loss of membrane during the inflammatory process, they measured the fixation of an antiglycophorin A antibody to the RBC membrane glycophorin A content. They observed an increased fixation of glycophorin A in RBCs from septic compared to nonseptic patients and to healthy volunteers [9]. These results excluded loss of the membrane and confirmed decreased RBC membrane SA content in septic patients facilitating links between antibody-glycophorin A. These results were in agreement with the results of Nieuwland et al. [10]. Interestingly, non-septic patients also had modifications of the RBC shape (more spherical) and decreased membrane SA content compared to RBCs from healthy volunteers. This group of patients is an “intermediate” population exhibiting a moderate inflammatory process that could alter the RBC membrane and shape. To explain these modifications of the RBC membrane SA content, Piagnerelli et al. suggested a possible increased activity and/or concentration of an enzyme, neuraminidase, which leaks SA during the inflammatory process [9]. Indeed, these authors also studied the desialylation process on one circulating protein—transferrin—in critically ill patients with and without sepsis [25]. In humans, circulating transferrin is represented by different glycoforms. The largest representative is tetrasialotransferrin, which binds 4 SA; the smallest representative is disialotransferrin, 2 SA, accounting for less than 1% of the concentration. Transferrin is considered as a “negative” acute phase protein, the concentrations of which decreases with the inflammatory process [26]. 

Because of the long half-life, approximately 16 days in humans, modifications of the SA pattern that were measured in patients are due to changes in a blood degradation rather than in synthesis. In patients admitted to the ICU with and without sepsis, these authors [25] observed increased concentrations of disialotransferrin in septic (18.3% [1.3–30.5]) compared to non-septic patients (0.7% [0.5–0.9]) and healthy volunteers (0.9% [0.5–1.1]; *P *< 0.05). They also measured increased concentrations of protein-bound SA and free SA concentrations in the septic patients. To prove that these modifications in SA metabolism occur rapidly in sepsis, the time course of the free SA concentrations was also measured in a model of septic shock induced by ligature and caecal perforation in sheep. An increased concentration of free SA was observed after 15 hours of sepsis in this animal model [25].

All these modifications could be explained by increased concentration and/or activity of neuraminidase. To demonstrate this hypothesis, the same group measured the neuraminidase activity in critically ill patients with and without sepsis [27]. They observed significantly increased neuraminidase activity in septic compared to nonseptic patients and healthy volunteers. To assess the effects of decreased RBC membrane SA content on deformability assessed by flow cytometry, these authors incubated neuraminidase from *Clostridium perfringens* at several concentrations (0.125, 0.25, and 0.5 U/mL) with RBCs from healthy volunteers and measured the free SA concentrations in the supernatant. After 2 hours of incubation with the higher concentrations of neuraminidase, these investigators observed the same modifications in RBC shape as had been observed in septic patients [9, 27]. Moreover, the RBC membrane contains a neuraminidase linked by a phosphatidylinositol link [28]. Incubation of RBCs from healthy volunteers with phosphatidylinositol phospholipase C (PIPLC) reproduced the same alterations in RBC shape and increased SA concentrations as observed in septic patients [27]. These data suggest a possible liberation of RBC membrane neuraminidase during sepsis. Nevertheless, several sources of neuraminidase have been reported: RBC membrane as described above and in other studies [29–31], WBCs [32–34], platelets [35], bacteria [36–39], and viruses [40, 41]. Indeed, some studies have shown that RBCs are able to recycle the free SA released by neuraminidase [42, 43] through a cytosolic sialate pyruvate lyase that specifically and reversibly catalyses the cleavage of SA to form N-acetylmannosamine and pyruvate [42, 43].

## 3. Links between RBC Alterations and the Microcirculation

Although several studies performed in animal models of sepsis and in human sepsis have reported alterations in RBC rheology [44–56], no studies have demonstrated the effects of altered RBCs on the microcirculation during sepsis. Several studies have reported the deleterious effects of transfused altered RBCs in sham animals. In sedated rats, Simchon et al. [57] showed the effects of transfused RBCs altered by incubation with glutaraldehyde and neuraminidase. These authors analysed the clearance of altered RBCs fixed by Cr^51^ and In^111^. They observed a decrease, by approximately 70%, of the altered RBCs in a few minutes in areas (liver, spleen, lungs, and kidneys) where the reticuloendothelial system was the most represented. Moreover, the blood flow neuraminidase RBC/control RBC ratio, measured by the microsphera technique (15 *μ*m, with a value of 1 as normal range), was markedly decreased for the spleen (0.4 ± 0.05), for the liver (0.66 ± 0.06), for the lungs (0.78 ± 0.03), and for the kidneys (0.78 ± 0.09). These data suggest a deleterious effect of neuraminidase-altered RBC deformability on blood flow [57].

Lux et al. [58] studied the effects of RBCs with deformability altered by different concentrations of glutaraldehyde on the rat pulmonary circulation. These authors observed an increased pulmonary arterial pressure related to the severity of the RBC deformability alterations. Baskurt in a rat model of isolated perfused leg demonstrated the same effect of altered RBCs on vascular resistance. They measured an increase in the resistance of up to 78% with the more altered RBC suspensions [59]. Cabrales [60] compared, in a model of isovolaemic haemodilution in the hamster, the effects of RBCs altered by glutaraldehyde, compared with Dextran 6% 70-kDA, and “fresh” RBCs, on the cutaneous microcirculation observed by the “window chamber.” He observed a decrease in flow and in diameter, especially in the arteriolar part of the microcirculation, with transfusion of altered compared to fresh RBCs, without modifications in blood viscosity. Interestingly, microvascular density was significantly decreased in the rats transfused with altered RBCs with decreased arteriolar, tissues, and venular PO_2 _[60].

There are several possible hypotheses to explain the effects of altered RBCs on tissue oxygenation. First, low flow rates lead to the depletion of arteriolar O_2_ and lowering of arteriolar blood PO_2_. The same effect was observed in the venular part of the circulation as a consequence of the lowered flow velocity after exchange with rigid RBCs leading to low PO_2_. Thus, the residence time of the RBCs within the vessel critically influences the amount of O_2_ that diffuses into the surrounding tissue, affecting O_2_ delivery to the capillary network. The altered RBC is associated in part with increased surface area-to-volume ratio, supporting the concept of decreased O_2_ uploading by rigid RBCs in the lung. This effect, in combination with the associated reduced arteriolar flow and functional capillary density, should explain why tissue and venular PO_2_ values are significantly reduced. Finally, decreased functional capillary density could be explained by microvascular vasoconstriction due to decreased ATP released by altered RBCs. Nevertheless, these hypotheses remain speculative, especially in septic conditions where decreased ATP release by altered RBCs has never been studied. 

The studies discussed above suggest deleterious effects of altered RBCs on the microcirculation, but the mechanisms have not really been evaluated. Moreover, these studies were not performed in septic models.

## 4. Relationship between Alterations in RBC Rheology, Microcirculation, and Outcome

Few investigations have studied RBC rheology and the microcirculation in septic patients. Donadello et al. [61] showed in 64 septic patients that worsened RBC deformability at day 3, as assessed by the laser-assisted optical rotational cell analyzer (LORCA, Mechatronics Instruments BV, AN Zwaag, Netherlands), was associated with a poor outcome. In contrast, RBC aggregation did not change over time in these patients [61]. Further studies investigating the microcirculation and RBC rheology in the same patients and the relationship of these aspects with mortality are needed in the future. 

In conclusion, components of the RBC membrane are modified during sepsis and may contribute to the observed alterations in RBC rheology. A better understanding of these processes could help identify strategies to improve RBC rheology and, thus, the microcirculation in this particular population of patients.

## Figures and Tables

**Table 1:** Main modifications of the RBC membrane observed during sepsis.

Membrane components	Model	Modifications reported	Effects	References
Proteins	Human RBCs	Membrane glycophorin A content increased during sepsis	Desialylation facilitates glycophorin A fixation	[9]
	Human serum of patients with meningococcemia	No changes in serum glycophorin A during the first 36 hours		[10]
	Mice RBCs with sepsis induced by caecal ligature and perforation	Increased band 3/*α*-spectrin ratio	Associated altered RBC deformability	[11]
		Phosphorylation of the band 3 and anion transporter capacity	No effects on anion transporter capacity	[12]
	Human RBCs	Decreased RBC proteins in septic and non-septic patients	No difference between septic and nonseptic patients	[13]

Lipids	Human RBCs	Increased membrane phosphatidylserine exposition	Increased entry of calcium → increased eryptosis ?	[14]
	Human RBCs	Increased membrane lipid peroxidation	Modifications of RBC lipid organization	[15]
	Rat RBCs	Controversial results on membrane lipid peroxidation	Effects on membrane fluidity?	[16, 17]

Carbohydrates	Human RBCs	Decreased sialic acid membrane content	Inverse relationship between spherical shape and decreased sialic acid membrane content. Stimulation of RBC glycolysis (increased lactate, 2,3-diphosphoglycerate)	[9]
